# Implications for Virtual Nursing Role Development in Acute Nursing Care: 24-Hour Time-and-Motion Study

**DOI:** 10.2196/87930

**Published:** 2026-05-08

**Authors:** Jennifer Sumner, Emily Hwee Hoon Chew, Sin Yee Peck, Janet Lam, Pauline Chong, Mun Kwong Ching, De Sheng Ong, Jacqueline Xiao Xuan Lau, Camille Keck, Abigail Ang, Jaminah Mohamed Ali, Hui Wen Lim, Anjali Bundele, Elya Chen, Carol Yap, Evelyn Ho, Amartya Mukhopadhyay, Alexander Wenjun Yip

**Affiliations:** 1 Medical Affairs – Research Innovation & Enterprise, Alexandra Hospital, 378 Alexandra Road, 159964, Singapore, 65 69082222, 65 69082222; 2 Department of Healthcare Redesign, Alexandra Research Centre for Healthcare in a Virtual Environment, Alexandra Hospital, Singapore; 3 Department of Nursing, Alexandra Hospital, Singapore; 4 Department of Data Science and Analytics, Alexandra Hospital, Singapore; 5 Yong Loo Lin School of Medicine, National University of Singapore, Singapore; 6 Division of Respiratory and Critical Care Medicine, National University Hospital, Singapore

**Keywords:** time and motion, nursing workflows, multitasking, inpatient, virtual nursing

## Abstract

**Background:**

Understanding current nursing workflows is essential to informing future workforce redesign strategies, including virtual nursing roles. However, granular insights into current nursing workflows over a 24-hour period and across different staff grades are lacking.

**Objective:**

This study aimed to (1) quantify how registered and enrolled nurses in the general acute medicine wards distribute their time across direct and indirect care tasks over a 24-hour period, (2) identify multitasking burdens and temporal distributions, and (3) identify opportunities for the development of a virtual nursing role.

**Methods:**

Using time-and-motion methodology, we observed registered and enrolled nurses in 3 general medicine wards over a 24-hour period between April 2024 and June 2024. We observed 3 task categories (administrative, communication, and bedside tasks), with multiple individual tasks monitored under each category. Multitasking (ie, the occurrence of 2 or more tasks concurrently) was also tracked. The checklist was piloted and refined before data collection.

**Results:**

We observed a total of 48 nursing shifts. During the daytime, registered nurses spent 70% (587/834 min) of their time on indirect care tasks compared with 54% (412/764 min) of the time for enrolled nurses. At night, the proportion of time spent on indirect care tasks decreased to 58% (410/705 min) for registered nurses and 39% (274/711 min) for enrolled nurses. During a 24-hour period, registered nurses spent 209 (SD 51.8) minutes multitasking in the day and 117 (SD 41.0) minutes at night, whereas enrolled nurses spent 152 (SD 54.7) minutes multitasking in the day and 110 (SD 75.9) minutes at night.

**Conclusions:**

These findings highlight opportunities for virtual nursing roles, which, if thoughtfully designed, may help redistribute indirect care tasks, reduce multitasking burden, and enhance overall efficiency without compromising care quality.

## Introduction

### Background

Health systems are facing a myriad of workforce challenges. Populations are aging, increasing the demand for care and the complexity of the care required [1]. Simultaneously, the workforce is shrinking; by 2030, the World Health Organization projects an estimated shortfall of 11 million health workers [2]. Furthermore, high-income nations find it increasingly challenging to recruit and retain home talent, relying on foreign workers to fill staffing gaps [3]. Challenges in recruitment and retention are further exacerbated by the increasing levels of staff dissatisfaction and burnout reported in recent years [4]. Collectively, these challenges raise significant questions about the sustainability of health care if no change occurs.

To address workforce challenges, there is an urgent need for role redesign and the careful application of technology to streamline processes and automate tasks. At Alexandra Hospital in Singapore, a new approach is being pioneered—the integration of a virtual workforce enabled through technology. Supported by the Ministry of Health, the hospital is investing in the development of remote or “virtual” nurses. In this context, virtual nursing refers to an emerging model of practice in which qualified nurses provide support to clinical team members remotely through digital technologies, thereby complementing bedside nursing. For example, virtual nurses may remotely monitor vital signs and fall risk, act as virtual discharge coordinators, participate in rounding through teleconsultation, or provide remote patient and caregiver education and staff supervision [5-8]. Additionally, the introduction of new virtual roles will provide more flexible work options such as working from home, which can support staff retention efforts [9]. Thus, virtual nursing is a promising model for workforce redesign.

To develop the virtual nursing workforce, understanding the status quo is important. Nurses are responsible for a range of direct (ie, involving direct interaction with patients) and indirect (ie, performed away from the patient) patient care tasks, including administering medication, monitoring patient conditions, coordinating with other health care providers, and managing administrative duties [10]. It is also well known that nurses handle diverse and often conflicting tasks, often leading to overwork and suboptimal practice [11] (eg, the duality of direct and indirect patient care tasks competing for attention). Furthermore, it has been widely reported that nurses frequently multitask (ie, perform multiple tasks simultaneously) throughout the day, which can create inefficiencies, increase the risk of errors, and reduce overall quality of care [12].

At the same time, nursing practice is fundamentally grounded in caring science, where the therapeutic nurse-patient relationship and presence are central to promoting healing and well-being [13]. However, growing administrative burden, competing indirect care demands, and multitasking can displace the relational aspects of care that form the essence of nursing. Therefore, understanding how nurses currently spend their time is essential not only for efficiency but to safeguard opportunities for caring practices. Role redesign—including virtual nursing—must be guided by the principle that technology should preserve and enhance nurses’ ability to deliver person-centered, relational care.

Despite this, much of the existing evidence base is qualitative, and there remains a gap in quantitative measurement. Where quantitative data exist, there is limited data capture on nighttime activities, the occurrence and patterns of multitasking over a 24-hour period, and the tasks undertaken by different staff grades (eg, registered nurses, who are qualified to dispense medications and instruct junior nurses, and enrolled nurses, who primarily handle patient care, such as bathing, feeding, and toileting) [10,11,14,15]. This study addresses these gaps by systematically mapping real-world nursing activity over a 24-hour cycle, examining the distribution of direct and indirect care tasks, identifying when multitasking occurs, and comparing activity patterns across staff grades. By doing so, this study provides empirical evidence to inform the design, timing, and scope of virtual nursing roles, identifying activities suitable for remote support without compromising patient-facing care.

### Aims

Our aims were as follows:

1. To quantify how registered and enrolled nurses in the general acute medicine wards distribute their time across direct and indirect care tasks over a 24-hour period
2. To identify multitasking burdens and temporal distributions
3. To identify opportunities for the development of a virtual nursing role

## Methods

### Overview

We used time-and-motion methodology to map and quantify the tasks and time spent on direct and indirect patient care in an inpatient setting. In brief, time and motion involves a period of continuous observation and timing of activities. This study is reported according to the Suggested Time and Motion Procedures checklist [16].

### Participants

This study was conducted at Alexandra Hospital in 3 general acute medicine wards. Ward sizes range from 19 to 33 beds, and each has several isolation beds where additional infection control measures are in place (eg, methicillin-resistant *Staphylococcus aureus* precautions). The typical nurse-to-patient ratio is 1 nurse for every 4 or 5 patients, with nurses opting for various full-day (12-hour) shifts or partial day shifts. A typical ward comprises a team of registered nurses (who are qualified to dispense medications and instruct junior nurses), enrolled nurses (who primarily handle patient care, such as bathing, feeding, and toileting), and a nurse manager who oversees ward operations. Each nurse has access to the electronic medical record (EMR) system through desktop computers or mobile phones (ie, Epic Rover).

As the roles of registered nurses and enrolled nurses are distinct, we observed both profiles through convenience sampling during a 3-month period. Inclusion criteria were being a full-time staff member and having worked on the wards for at least 6 months. Exclusion criteria were basic care assistants, part-time workers, or those with less than 6 months of experience on the wards. The month before data collection commenced, briefing sessions were held with the nurse managers to introduce the research project and seek their approval. Observers (health service research staff) had no direct relationship with the nursing staff being observed.

### Data Collection

The initial checklist of observation items was developed through consultation with the ward managers and comprised 3 overarching domains (administrative, communication, and bedside tasks), 8 task subcategories (activities of daily care, patient communication, clinical care, administrative tasks, staff communication, staff education, ward observation, and break time), and 27 individual task codes, mapped in Table 1. The checklist was piloted and refined during observations between November 29, 2023, and December 8, 2023, before the actual data collection commenced. A second pilot was conducted from April 9 to 12, 2024, to standardize the observation process and train the 10 observers. Tasks were observed and tracked concurrently to capture periods of multitasking (ie, when 2 or more tasks were performed at the same time). For example, a communication task could be timed at the same time as a bedside care task. In our study, transition periods (short intervals between the end of one task and the start of another) were recorded as part of the subsequent task whenever the transition was functionally inseparable from task initiation (eg, walking from a workstation to a patient’s bedside to begin care).

Data collection consisted of continuous nurse observation over 1 complete work shift (8:30 AM-8 PM or 8 PM-8:30 AM). Observations were conducted between April 15, 2024, and June 24, 2024. A roster was created in Python (version 3.8; Python Software Foundation) randomly allocating observers across different days of the week and weekend slots across the 3 wards. We used the Time Capture Tool TimeCaT on an iPad (Apple Inc) to track activities. TimeCaT is a validated tool frequently used in observations of health care processes [17]. Tasks are entered into the app a priori, and observers select the appropriate task as it occurs, activating a timer. Observers can also add notes to specific data points. During data collection, we also captured bed occupancy, the number of patients discharged, and the number of bed days.

**Table 1.** Direct and indirect care tasks, subcategories, and individual task codes.

Task categories and subcategories	Task codes
Direct care tasks	
Supporting activities of daily living	Ambulation Bathing Changing bedding Diapering or assisting with toileting Assisting with food and drink
Patient communication	Nurse to patient Meal ordering Attending patients’ call bell
Clinical care	Vital sign taking Laboratory samples (collection and sending) Other assessments and procedures Medication handling Patient comfort Patient transfer (diagnostics) Waste disposal (clinical and nonclinical waste)
Indirect care tasks	
Administrative	Electronic medical record entry Record viewing (no data entry) Nonclinical administrative tasks Discharge or admission
Staff communication	In-person staff member to staff member Remote staff member to staff member Remote staff member to caregiver Handover Team meetings
Staff education	Staff education
Ward observation	Observing patients
Break time	Break

### Ethical Considerations

This study was reviewed and approved by the National University of Singapore Ethical Review Board (NUS-IRB-2023-327). A waiver for consent was obtained from the National University of Singapore Institutional Review Board. Data were anonymous and analyzed at the aggregate level. Participants were not individually compensated.

### Data Analysis

Before analysis, each observation data file was cleaned. TimeCaT includes a note function that allows observers to comment on specific data points. Each note was reviewed, and revisions were made to the data if required (eg, removal of tasks that were erroneously timed or relabeling of incorrect observations). We then mapped the observed tasks to direct and indirect care tasks, creating subcategories within each domain. Direct care tasks involved direct interaction with the patient (eg, bathing and procedures), and indirect care tasks were those that were performed away from the patient (eg, documentation and staff communication) but on behalf of the patient (Table 1).

Time-and-motion data were analyzed in multiple steps. To calculate the average time spent on each task, we summed the total time spent on each task across all observations, including instances in which the task was not performed (ie, zero time), and divided it by the total number of observations. This produced the average task load time for a typical day or night shift. We then calculated the total task load time for a shift by summing all average task times and used this value to determine the proportion of time spent on each task as a fraction of the total task load time. To analyze the data by hour, each observed task was assigned to a 1-hour “time bin.” For example, a task performed from 9:01 AM to 9:20 AM was categorized under the 9 AM to 10 AM time bin and recorded as 19 minutes. If a task spanned multiple time bins, its duration was divided accordingly. This approach enabled accurate tracking of task distribution across the 24-hour period. We also measured multitasking by calculating the total time during which 2 or more tasks were performed simultaneously (eg, communication and administrative tasks). Periods of no activity were excluded from the analysis. All data processing and analyses were conducted using Python (version 3.8) and Stata (version 19; StataCorp LLC).

### Validity and Reliability

The observation checklist was tested and optimized over 6 separate pilot observations (a total of 17.5 hours) between November 29, 2023, and December 8, 2023. Prior to the actual data collection, a training session was conducted (April 8, 2024) to introduce the team of observers to the process and address any initial questions. A set of training observations was then conducted in pairs from April 9 to 12, 2024, to familiarize the observers with the process, standardize coding of observed tasks, and ensure that the checklist was complete. The team met 4 times during this period to discuss coding alignment and resolve any questions. Interrater agreement was assessed during the pilot phase of the study [18]. As tasks across the 3 domains (administrative, communication, and bedside) could occur concurrently, the Cohen κ statistic and percentage of agreement were calculated separately for each domain. For each observer pair, a 30-minute observation period was analyzed. The observational data were divided into 30-second intervals, and agreement between coders was assessed based on the task assigned to each interval. κ values ranged from 0.62 to 0.69, indicating substantial agreement, whereas the percentage of agreement ranged from 78% to 84%, reflecting good consistency between coders. Formal data collection commenced on April 15, 2024.

## Results

### Overview

During the study period, we observed 48 shifts, including a total of 30 nurses during the day (8:30 AM-8 PM; n=22, 73.3% registered nurses and n=8, 26.7% enrolled nurses) and 18 nurses at night (8 PM-8:30 AM; n=14, 77.8% registered nurses and n=4, 22.2% enrolled nurses). The total active task observation time for registered nurses was 164 hours and 29 minutes, and for enrolled nurses, it was 47 hours and 22 minutes. During the observation period, bed occupancy ranged from 71% to 90%, and 1044 patients were discharged from the wards, representing 5559 bed days.

### Task Load Over a 24-Hour Period

**Figure 1.**
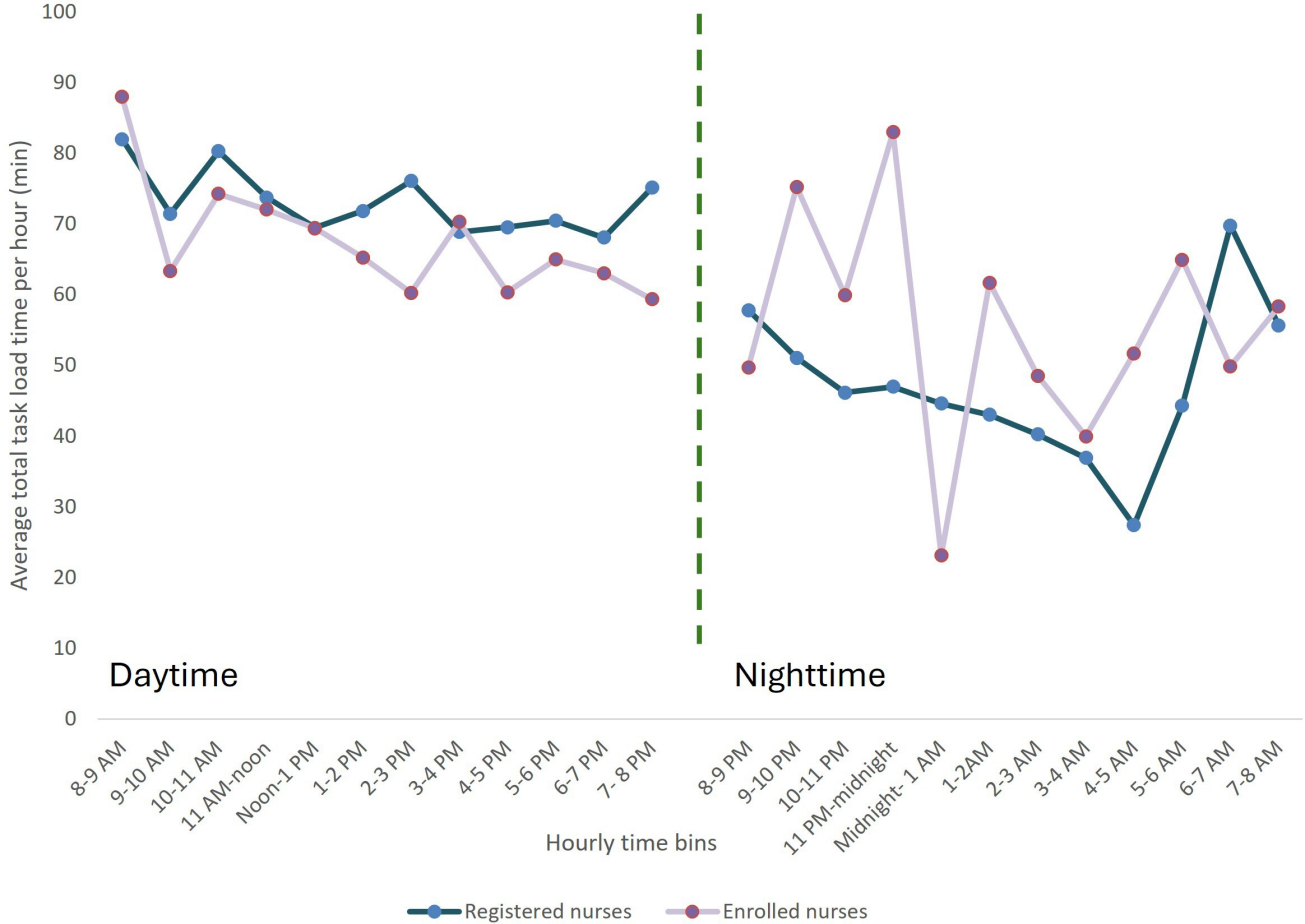
Total task load (minutes) by hour for a 24-hour period (task load by hour exceeds 60 minutes due to multitasking) for registered nurses (blue) and enrolled nurses (pink).

Task load varied over the course of 24 hours, with higher variability at night—particularly for enrolled nurses (Figure 1). During the day, the highest peak in task load for registered and enrolled nurses was between 8 AM and 9 AM, whereas the lowest task load was between 6 PM and 7 PM for registered nurses and between 7 PM and 8 PM for enrolled nurses. In the night shift, the highest task load peak was between 6 AM and 7 AM for registered nurses and between 11 PM and midnight for enrolled nurses. Conversely, the lowest task load was observed from 4 AM to 5 AM for registered nurses and midnight to 1 AM for enrolled nurses.

### Task Type: Direct and Indirect Nursing Care Tasks

**Figure 2.**
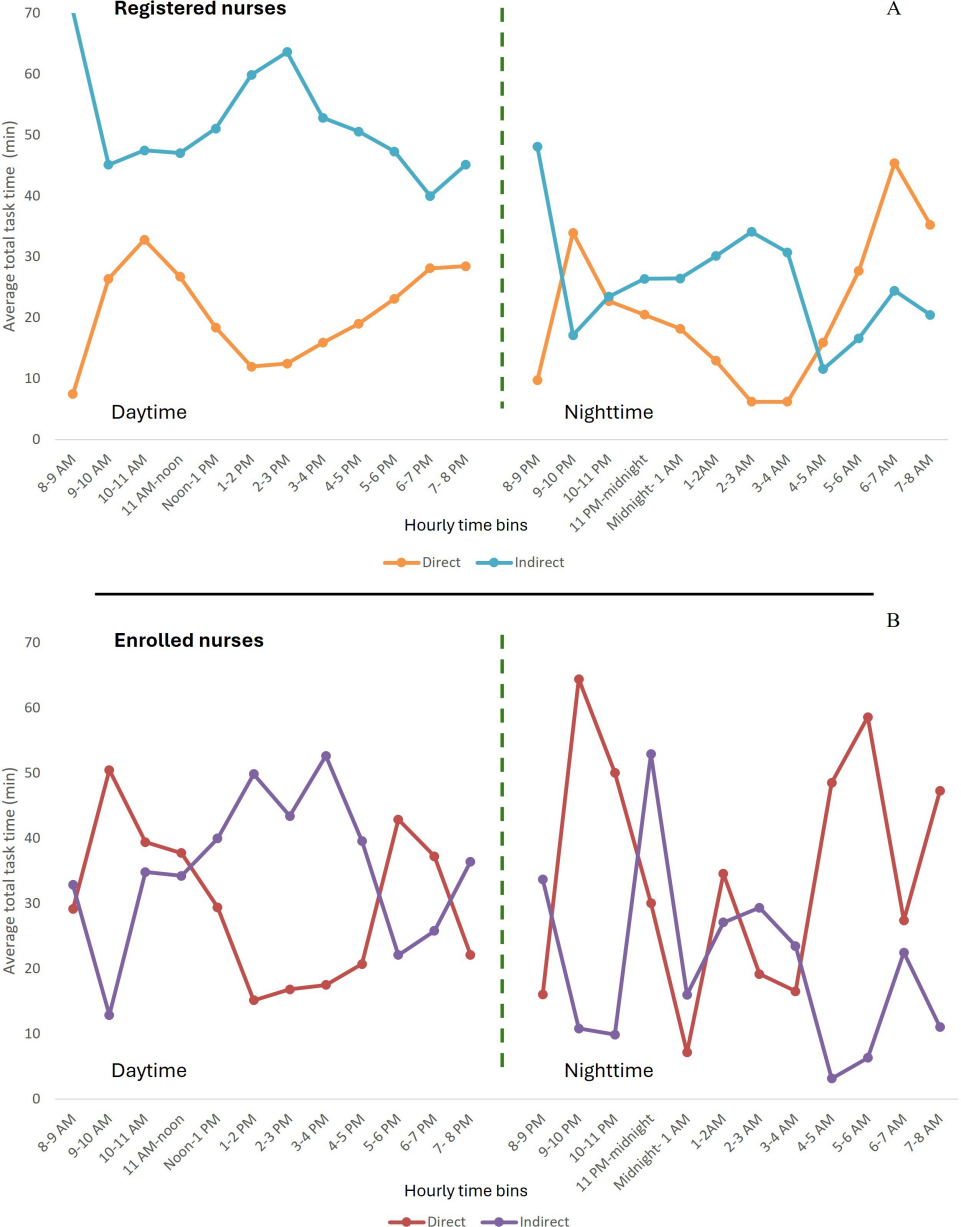
Direct and indirect total task load (minutes) by hour for (A) registered nurses and (B) enrolled nurses.

During the day, registered nurses spent an average of 30% (248 min; SD 74.6) of their time on direct clinical care and 70% (587 min; SD 98.9) of their time on indirect care tasks (Figure 2 and Table 2). Enrolled nurses, in contrast, spent an average of 46% (351 min; SD 52.5) of their time on direct care tasks and 54% (411 min; SD 73.2) of their time on indirect care tasks. At night, the proportion of time spent on direct care tasks increased for both groups: registered nurses spent an average of 42% (295 min; SD 78.9) of their time on direct care tasks, whereas enrolled nurses spent 61% (436 min; SD 98.5) of their time on direct care tasks, with the remainder dedicated to indirect care tasks (410 min, 58%; SD 67.2) for registered nurses, and 39% (275 min; SD 31.7) for enrolled nurses.

For registered nurses, indirect tasks dominated, with a consistently higher average total task load during the daytime (Figure 2). Direct task load typically remained at less than 30 minutes per hour, with a peak between 10 AM and noon and between 6 PM and 8 PM. At night, there was a noticeable drop in time spent on indirect tasks, and time spent on direct care tasks gradually decreased until 3 AM. From 3 AM onward, direct care tasks increased, peaking at 6 AM. For enrolled nurses, time spent on direct care tasks tended to dominate over time spent on indirect care tasks for much of the day, especially in the early morning and late afternoon, with a dip in the middle of the day. At night, there was considerable variability in both direct and indirect care tasks. There were notable spikes in direct care tasks (eg, around 11 PM-1 AM and 5 AM‐7 AM) and indirect care tasks (eg, 11 PM-midnight), suggesting time-specific care demands.

For registered nurses, medication handling (88/834 min, 10%), procedures (46/834 min, 5%), and patient communication (46/834 min, 5%) were the largest contributors to direct care task load during the day (percentage of total task time), with no change at night (Table 2). Staff communication (134/834 min, 16%), data entry into the medical record system (117/834 min, 14%), and record viewing (104/834 min, 12%) were the largest contributors to indirect care task load. At night, data entry and record viewing remained as the top tasks, but time spent on staff communication decreased, whereas handover time increased from 5% (38/834 min) during the day to 8% (59/705 min) at night.

For enrolled nurses, procedures (85/764 min, 11%), assisting with toileting or diaper changes (57/764 min, 8%), and patient communication (57/764 min, 8%) were the largest contributors to direct care task load during the day (percentage of total task time), with no change at night. In addition, the proportion of task time spent on vital sign taking increased from 4% (33/764 min) during the day to 12% (82/711 min) at night. Staff communication (107/764 min, 14%), data entry into the medical record system (90/764 min, 12%), and record viewing (60/764 min, 8%) were the largest contributors to indirect care task load during the day. At nighttime, data entry and record viewing remained high contributors to task time, but staff communication decreased to 2% (11/711 min), whereas observation of patients in the ward became the third largest contributor at 7% (52/711 min).

**Table 2.** Average time taken (minutes) on tasks during the day or night for registered nurses (RNs) or enrolled nurses (ENs).

Task category, subcategory, and task codes	Time taken on tasks during a day shift (min), mean (SD)		Time taken on tasks during a night shift (min), mean (SD)	
	RN	EN	RN	EN
Direct care tasks				
Supporting activities of daily living				
Ambulation	2 (2.7)	10 (7.7)	3 (4.7)	7 (2.0
Bathing	13 (17.2)	30 (20.2)	2 (5.7)	7 (6.0)
Changing bedding	4 (5.2)	12 (7.7)	1 (1.8)	2 (1.5)
Diapering or assisting with toileting	12 (13.3)	57 (30.2)	33 (24.7)	103 (65.7)
Assisting with food and drink	9 (11.5)	19 (14.0)	3 (4.2)	18 (7.5)
Patient communication				
Nurse to patient	46 (22.6)	57 (26.7)	62 (41.4)	99 (112.2)
Meal ordering	0.4 (1.1)	4 (6.4)	1 (2.3)	2 (1.4)
Attending the patient call bell	1 (2.6)	2 (2.0)	2 (3.0)	4 (1.3)
Clinical care				
Vital sign taking	5 (6.4)	33 (12.4)	17 (12.9)	82 (23.2)
Laboratory samples	9 (13.1)	1 (2.1)	26 (20.1)	3 (5.8)
Other assessments and procedures	46 (24.7)	85 (25.6)	41 (22.8)	87 (16.5)
Medication handling	88 (44.3)	11 (13.3)	96 (34.7)	4 (3.3)
Patient comfort	4 (3.0)	14 (8.4)	5 (5.2)	4 (3.4)
Patient transfer (diagnostics)	5 (12.0)	8 (17.4)	0.3 (1.2)	5 (10.3)
Waste disposal (clinical and nonclinical waste)	3 (2.1)	9 (2.8)	3 (2.4)	10 (4.2)
Indirect care tasks				
Administrative				
Electronic medical record data entry	117 (37.2)	90 (38.6)	92 (32.3)	59 (23.1)
Record viewing (no data entry)	104 (41.9)	60 (43.8)	92 (39.3)	53 (14.2)
Nonclinical administrative tasks	24 (21.3)	17 (18.4)	9 (14.7)	7 (3.7)
Discharge or admission	19 (16.9)	4 (4.3)	8 (13.6)	3 (2.7)
Staff communication				
In-person staff member to staff member	134 (36.6)	107 (18.4)	46 (41.0)	11 (9.5)
Remote staff member to staff member	22 (11.2)	2 (1.4)	8 (6.6)	0 (0)
Remote staff member to caregiver	1 (1.8)	0 (0)	0.4 (1.0)	0 (0)
Handover	38 (30.5)	16 (15.1)	59 (23.9)	18 (21.4)
Team meetings	22 (14.6)	11 (16.4)	10 (7.1)	10 (14.7)
Staff education				
Staff education	36 (35.5)	4 (6.2)	3 (5.8)	0.4 (0.8)
Ward observation				
Observing patients	6 (8.3)	8 (5.3)	41 (70.9)	52 (69.7)
Break time				
Break	64 (32.2)	93 (22.4)	40 (27.3)	61 (3.2)

### Multitasking Load

**Figure 3.**
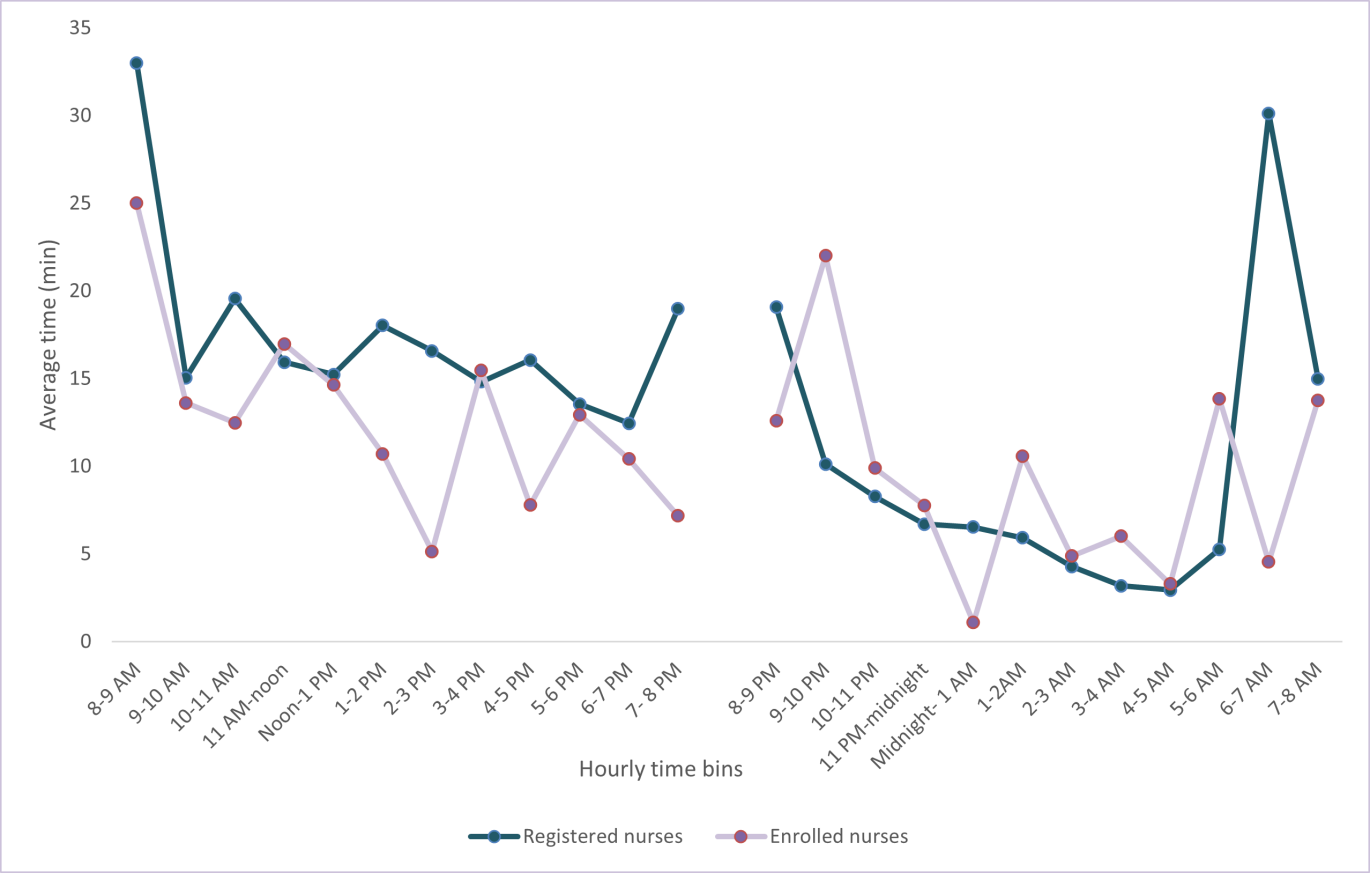
Average time (minutes) spent multitasking in each hour for a 24-hour period by registered nurses (blue) and enrolled nurses (pink).

During a 24-hour period, registered nurses typically spent 209 (SD 51.8) minutes multitasking in the day and 117 (SD 41.0) minutes at night. For enrolled nurses, 152 (SD 54.7) minutes were spent multitasking in the day and 110 (SD 75.9) minutes at night. The frequency of occurrence varied over the course of the day (Figure 3). During the daytime, the period with the highest multitasking load was between 8 AM and 9 AM for registered nurses (33, SD 14.6 min) and enrolled nurses (25, SD 11.7 min), and the lowest multitasking load was observed between 6 PM and 7 PM for registered nurses and between 2 PM and 3 PM for enrolled nurses. At nighttime, the period with the highest level of multitasking was 6 AM to 7 AM for registered nurses (30, SD 33.1 min) and 9 PM to 10 PM for enrolled nurses (22, SD 16.3 min).

## Discussion

### Principal Findings

To address ongoing workforce challenges, there is a pressing need for role redesign and the careful application of technology to support nursing practice. This study provided empirical insights into how nurses in general medicine wards spend their time across a 24-hour period, including the distribution of direct and indirect care activities and the occurrence of multitasking. This study is part of a wider program of work to inform how virtual nursing roles can be designed and integrated into ward workflows by identifying which tasks may be amenable to remote support and during which times support may be most beneficial.

Our findings show that nurses spent only 30% (247/834 min) to 46% (352/764 min) of their time on direct clinical care; the remainder was dedicated to indirect care tasks such as documentation and coordination. Task loads frequently exceeded 60 minutes of activity per hour, indicating significant multitasking and overlapping responsibilities. Such patterns reflect recognized inefficiencies in inpatient workflows, where competing demands require nurses to switch rapidly between tasks, increasing cognitive load and potential for error. We also observed predictable peaks in indirect care duties—particularly around early-morning and evening shift transitions—where administrative and communication tasks accumulated. In contrast, night shift patterns demonstrated marked variability, with periods of low activity followed by compressed workloads toward the end of the shift, when cumulative fatigue is likely to be highest. These specific patterns represent risk-prone periods within the nursing workday.

From a service design perspective, these findings suggest that virtual nursing support may be particularly beneficial when targeted to predictable high-risk periods, such as shift transitions and late-night hours, rather than implemented as a constant or uniformly distributed resource. Our data show that documentation and coordination activities cluster at these transition points, creating periods where clinical sensemaking and narrative continuity may be compressed or fragmented. While often treated as discrete tasks, documentation and handover are essential to a nurse’s understanding of patient care and response over time. Thus, the design of virtual nursing roles should support clinical sensemaking by assisting with preparatory documentation, information synthesis, and record completion while preserving the primary nurse’s responsibility for clinical interpretation, evaluation, and relational care. In this way, virtual nursing supports continuity of care rather than displacing the ground nurse’s knowledge of the patient.

Our analysis showed that nurses frequently experienced task loads exceeding 1 hour of activity per observed hour, reflecting substantial multitasking and the fragmented nature of nursing work [10,11,14,15]. While some multitasking may improve efficiency, excessive cognitive load increases the likelihood of errors and reduces care quality, underscoring the need to reconsider how workload is organized [19,20]. By mapping when peaks in documentation, coordination, and multitasking occur, we highlight concrete opportunities for virtual nursing integration. For example, indirect care tasks that cluster around shift handover—such as preparing documentation, updating EMR entries, and coordinating care plans—could be supported by virtual nurses operating remotely. Importantly, the high degree of task concurrency observed suggests that virtual nursing should prioritize offloading cognitively competing tasks (eg, documentation and coordination) rather than introducing additional parallel interactions that could further fragment attention.

Night shifts exhibited greater variability, particularly for enrolled nurses, with pronounced peaks and a late-shift surge, when fatigue is likely to impair performance and elevate error risk [21,22]. Prior studies have raised similar concerns, noting that staffing levels during night shifts often fail to meet patient needs, leading to increased stress and task compression or even unresolved tasks [23-25]. Although interventions such as scheduled napping and optimized lighting can mitigate fatigue [26-28], they do not address the underlying workload imbalance. These patterns point to specific opportunities for virtual nursing support, such as documentation, discharge preparation, remote monitoring, and assisting junior staff, to relieve peak pressures and enhance workflow continuity [29-31]. Aligning virtual nursing activity with these high-pressure windows offers a targeted approach to smoothing workflow peaks, reducing multitasking burden, and supporting safer, more sustainable practice.

Analysis of the types of nursing activities revealed that a substantial proportion of time was spent on indirect care tasks, particularly EMR documentation, by both registered and enrolled nurses. This aligns with extensive literature showing that administrative workload, especially documentation, is a major contributor to reduced direct care time, job dissatisfaction, and burnout [10,14,20,32,33]. From the perspective of the theory of human caring by Watson [13], excessive administrative demands diminish nurses’ capacity to engage in the relational, patient-centered interactions that underpin caring-healing practices, such as being fully present, developing trust, and supporting patients’ emotional and existential needs. Our findings reinforce these concerns by showing that administrative peaks occur at predictable times, particularly during shift changes, thereby reducing opportunities for therapeutic engagement. Rather than treating documentation reduction as a generic goal, our findings indicate that documentation support is most critical during temporally concentrated peaks—particularly at shift transitions, where administrative demands can displace opportunities for patient-facing care. These patterns highlight clear opportunities for workflow redesign, including improved handover protocols, EMR-integrated automation tools, and shifting routine documentation to virtual care teams or dedicated virtual administrative assistants. Early evaluations of virtual nursing models show promise in reducing documentation burden, improving workflow efficiency, and enhancing staff satisfaction [34-37]. Our data help identify where such support would be most impactful, ultimately enabling bedside nurses to devote greater attention to the caring practices central to the nursing profession.

Taken together, these insights offer timely evidence to guide the development of virtual nursing roles within inpatient workflows. Virtual nurses, equipped with real-time access to EMRs, vital sign dashboards, secure messaging, and telepresence tools, could assume a range of indirect care tasks—documentation, discharge coordination, remote education delivery, and virtual rounding [29-31,35-38]. Importantly, we should tailor support by staff role: for registered nurses, reducing documentation and coordination workload allows for greater focus on complex clinical care, whereas for enrolled nurses, virtual teams could assist with supervisory or routine administrative tasks [29-31,35-38]. Over time, this infrastructure also provides a foundation for automating selected processes with artificial intelligence–enabled tools, contributing to more scalable and sustainable workforce models.

At the same time, it is essential that the ethical considerations of virtual nursing are taken into account. Redistributing nursing work to virtual roles raises important ethical considerations related to professional responsibility, accountability, and the nurse-patient relationship. While indirect care activities may be performed remotely, clinical judgment, evaluation of patient responses, and ownership of care outcomes must remain with the primary nurse responsible for the patient. Therefore, our findings highlight the importance of designing virtual nursing models that reduce cognitive and administrative burden without fragmenting accountability or distancing nurses from patient care, ensuring that virtual support enhances—rather than replaces—the ethical commitments embedded in bedside nursing practice.

### Limitations

Our direct observations allowed us to gather unbiased data on nursing processes and report objectively on time use, overcoming the limitations of qualitative interview or survey data, which may be prone to bias. Our observation checklist was comprehensive, allowing for a fine-grained analysis of the task load. However, limitations must be acknowledged. We cannot ignore the Hawthorne effect, where staff may alter their behavior while being observed. We attempted to minimize this by briefing nursing teams and taking multiple sampling points over a 3-month period. There may also have been issues with observer variability. To minimize this risk, we conducted pilot work and training sessions with each observer to standardize the process. Observers were also encouraged to discuss issues and ask for clarifications during regular team meetings. Finally, our sampling took place during a 3-month period, which may not accurately reflect the average task load across the year.

### Conclusions

This time-and-motion study documents the substantial and complex workload experienced by inpatient nursing staff over a 24-hour period across different nurse roles. The data reveal consistently high task demands, frequent multitasking, and pronounced peaks and troughs in workload throughout the day and night. These patterns suggest significant cognitive strain, with potential consequences for staff well-being and patient safety. These insights can inform the design of virtual nursing models or other technology-enabled workforce innovations. Future research should test and evaluate these models through staged implementation and outcome assessment, ensuring that virtual and hybrid care teams are safe, effective, and responsive to the realities of inpatient workflows.

## Abbreviations

EMR: electronic medical record
